# Risk assessment analysis for maternal autoantibody-related autism (MAR-ASD): a subtype of autism

**DOI:** 10.1038/s41380-020-00998-8

**Published:** 2021-01-22

**Authors:** Alexandra Ramirez-Celis, Martin Becker, Miriam Nuño, Joseph Schauer, Nima Aghaeepour, Judy Van de Water

**Affiliations:** 1 Department of Internal Medicine, Division of Rheumatology, Allergy, and Clinical Immunology, One Shields Avenue, University of California, Davis, CA 95616 USA; 2 Department of Anesthesiology, Pain, and Perioperative Medicine, Stanford University, Palo Alto, CA 94305 USA; 3 Department of Pediatrics, Stanford University, Palo Alto, CA 94305 USA; 4 Department of Biomedical Data Sciences, Stanford University, Palo Alto, CA 94305 USA; 5 Department of Public Health Sciences, Division of Biostatistics, One Shields Avenue, University of California, Davis, CA 95616 USA

**Keywords:** Predictive markers, Autism spectrum disorders

## Abstract

The incidence of autism spectrum disorder (ASD) has been rising, however ASD-risk biomarkers remain lacking. We previously identified the presence of maternal autoantibodies to fetal brain proteins specific to ASD, now termed maternal autoantibody-related (MAR) ASD. The current study aimed to create and validate a serological assay to identify ASD-specific maternal autoantibody patterns of reactivity against eight previously identified proteins (CRMP1, CRMP2, GDA, NSE, LDHA, LDHB, STIP1, and YBOX) that are highly expressed in developing brain, and determine the relationship of these reactivity patterns with ASD outcome severity. We used plasma from mothers of children diagnosed with ASD (*n* = 450) and from typically developing children (TD, *n* = 342) to develop an ELISA test for each of the protein antigens. We then determined patterns of reactivity a highly significant association with ASD, and discovered several patterns that were ASD-specific (18% in the training set and 10% in the validation set vs. 0% TD). The three main patterns associated with MAR ASD are CRMP1 + GDA (ASD% = 4.2 vs. TD% = 0, OR 31.04, *p* = <0.0001), CRMP1 + CRMP2 (ASD% = 3.6 vs. TD% = 0, OR 26.08, *p* = 0.0005) and NSE + STIP1 (ASD% = 3.1 vs. TD% = 0, OR 22.82, *p* = 0.0001). Additionally, we found that maternal autoantibody reactivity to CRMP1 significantly increases the odds of a child having a higher Autism Diagnostic Observation Schedule (ADOS) severity score (OR 2.3; 95% CI: 1.358–3.987, *p* = 0.0021). This is the first report that uses machine learning subgroup discovery to identify with 100% accuracy MAR ASD-specific patterns as potential biomarkers of risk for a subset of up to 18% of ASD cases in this study population.

## Introduction

Autism spectrum disorder (ASD) is characterized by social and behavioral impairments, along with restricted interests and repetitive behaviors. In 2018, the CDC estimated that 1 in 59 children are affected in the USA [1], making ASD an important health concern and a substantial socioeconomic burden for affected families and the healthcare system [2, 3].

We previously described specific maternal autoantibody reactivity against seven proteins highly expressed in the developing brain including collapsin response mediator proteins 1 and 2 (CRMP1, CRMP2), guanine deaminase (GDA), lactate dehydrogenase A and B (LDHA, LDHB), stress-induced phosphoprotein-1 (STIP1), and Y-box binding protein 1 (YBOX). These earlier studies identified autoantibody reactivity against these antigens by western blot (WB) in plasma from mothers whose children were diagnosed with ASD (23%) with only 1% in the typically developing group (TD) [4]. More recently, we discovered an additional target autoantigen, neuron-specific enolase (NSE) [5]. Further, we performed autoantibody epitope mapping for each of the eight antigens and found peptide sequences recognized only by maternal samples from the ASD group [5, 6]. We have termed this subtype of ASD as Maternal Autoantibody-Related (MAR) autism.

In the current study, our primary goal was to improve upon our previous findings through the development of a highly accurate and specific ELISA test for the assessment of maternal autoantibody reactivity against the eight antigens, thus enabling the ability to predict the risk of having a child with ASD. To achieve this goal, we used machine learning (ML) techniques to identify and evaluate the precision for the patterns of reactivity to the eight autoantigens. Success in the current study will allow the future development of this technology and exploration of these autoantibody patterns as predictors of an ASD diagnosis.

## Materials and methods

### Study subjects

Biologic samples for this study were from mothers enrolled in the Childhood Autism Risks from Genetics and Environment (CHARGE) study [7]. This project included mothers of children diagnosed with ASD (*n* = 450) and of children selected from the general population and evaluated as neurotypical (typically developing, TD; *n* = 342). The participants provided written consent and fulfilled the recruitment and eligibility criteria. All the children underwent diagnostic evaluation including medical, social, and cognitive assessment as previously described [7, 8]. The demographic information related to these samples is shown in Supplementary Table 1.

### Sample collection and preparation

Maternal blood was collected in citrate dextrose (BD Diagnostic) and plasma was separated, labeled, aliquoted, and stored at −80 °C. Prior to use, samples were thawed at room temperature (RT), vortexed, and centrifuged at 13,000 RPM for 10 min.

### Experimental groups

The samples were randomly divided into two experimental groups: (1) The training set (*n* = 375; ASD = 206, TD = 169), to determine the reactivity patterns and evaluate the association between reactivity and diagnosis (ASD), and (2) the validation set (*n* = 418; ASD = 244, TD = 174) which served to corroborate the patterns discovered using the training set and the association of those patterns with an ASD diagnosis.

### Proteins

An important aspect of the recombinant proteins used to build this assay was the removal of the His-Tag from the antigens due to the nonspecific binding by human plasma samples to His-Tag used in prokaryotic expression systems. The tag-less proteins CRMP1 #MBS7074427 and LDHA #MBS949692 were from MyBioSource (San Diego CA), GDA # NBP2-49692, LDHB #NBP2-49694, NSE #NBC1-18342, and STIP1 #NBP2-49685 were from Novus Biologicals (Centennial, CO), while CRMP2 and YBOX were custom made by Expression Systems using a baculovirus system (Davis, CA).

### Enzyme-linked immunosorbent assay (ELISA)

Autoantibody reactivity of plasma samples against protein antigens was determined by ELISA and corroborated by WB using commercially available proteins as previously described [5]. The protein concentration and plasma sample dilutions were optimized for each antigen for both assays. In summary, microplates were coated with 100 μl of antigen (1.5–3 µg/µl) in carbonate coating buffer pH 9.6, incubated overnight at 4 °C, washed four times with Phosphate Buffered Saline Tween-20 (PBST) 0.05%, and blocked with 2% Super Block (Thermo Scientific, Rockford, lL) for 1 h at RT. The plasma samples were diluted 1:250–1:1000, and run in duplicate. Following dilution, 100 µl of the diluted sample was added to each well, incubated for 1.5 h, washed 4 times in PBST 0.05%, and then washed four times with (PBST) 0.05%, and incubated with goat antihuman IgG-HRP IgG (Kirkegaard & Perry Laboratories, Inc., Gaithersburg, MA) diluted 1:10,000 for 1 h. The plates were then washed four times with (PBST) 0.05%, and detection was performed by adding 100 µl of BD optEIA liquid substrate for ELISA (BD Biosciences, San Jose, CA). After 4 min, the reaction was stopped with 50 µl of 2N HCl. The absorbance was measured at 490–450 nm using an iMark Microplate Absorbance Reader (Biorad, Hercules, CA, USA). Of note, we only examine IgG reactivity since it is the only isotype able to cross the placenta, which is a key component of the MAR ASD mechanism.

### Statistical analysis

#### Receiver operating characteristic (ROC) curve

For the ELISA assay, a positive cutoff value for reactivity to each antigen was determined using an ROC curve as previously described [5]. Youden’s index was used to calculate the specific numerical threshold cutoff for each protein, for each set after plate–plate normalization. The cutoff was optimized for each set and antigen [9, 10]. The seven control-positive samples that were used to create the ROC curves were not included in the pattern discovery analysis.

#### Pattern identification

We identified as positive samples that were reactive for a combination of 2 or more of the 8 antigens and that were perfectly specific for ASD diagnosis and compared these patterns of reactivity between TD and ASD groups by Fisher Exact test (*P* < 0.05). To determine if the association of specific patterns of reactivity with ASD outcome was greater than would be obtained by chance, we conducted a permutation analysis [11, 12]. All permutation analysis was done using SAS^®^ software version 9.4 (Cary, NC).

To detect high-precision ASD indicator patterns, we employed methods from subgroup analysis and exceptional model mining. In particular, we applied a depth-first search algorithm to identify patterns based on a family of interestingness measures that judges the quality of pattern by weighing its precision against its support [13, 14]. We focused on patterns with high precision by setting the weighting parameter *a* = 0.01. For the current study, we limited the length of the patterns to three. The results of this analysis are shown in Supplementary Tables 2, 3. To visualize the relations between patterns, we built a dependency network, where each pattern is represented by a binary vector based on the instances it covers. Based on this, the patterns are then embedded into a two-dimensional space via PCA. The patterns are connected based on the relationship between their descriptions and covered instances. This enables deeper insights into which patterns may be redundant with regard to their descriptions or related with regard to the samples they cover. Finally, we evaluated several predictive models, one based directly on the patterns we have found as well as several multivariate models that are based on state-of-the-art ML approaches. These models were trained and tested on the previously defined training and validation set. Parameter optimization was solely performed on the training set. For the pattern-based algorithm, we extracted top-k patterns from the training set and predicted ASD for a new subject if any of these patterns matched this subject’s reactivity profile.

#### ADOS severity score correlation

To investigate if autoantibody reactivity was relevant to the Autism Diagnostic Observation Schedule (ADOS) severity score, we analyzed 254 ASD samples that had autoantibody reactivity to at least one antigen. We used a least absolute shrinkage and selection operator (LASSO) ordinal logistic regression model to determine important autoantibody–antigen combinations that describe the ADOS severity score. LASSO helped increase model interpretability by eliminating irrelevant autoantibody antigens that were not associated with ADOS severity score and reduced model overfitting. We assessed the model predictive performance using the Schwarz Bayesian criterion and cross validation. Once relevant interactions were selected, we used a generalized ordinal logistic regression model for ordered outcome (1–10). Computing was done in SAS^®^ software version 9.4 (Cary, NC).

## Results

### Autoantibody reactivity against fetal brain antigens

As mentioned previously, we divided the samples into two experimental sets (1) the training set to determine autoantibody reactivity combinations associated with an ASD outcome, and (2) the blinded validation set to test the accuracy of patterns identified in the training set for prediction of ASD. Supplementary Table 4 shows a summary of maternal antibody reactivity to the eight target autoantigens.

For the training set, we found that of 375 samples, 229 had autoantibody reactivity to at least one antigen (ASD = 134, 65% and TD = 96, 57%; *p* = 0.1108), demonstrating that autoantibodies against brain antigens are not individually correlated with diagnosis, as previously observed [4]. Additionally, we did not observe any differences in the reactivity level (low, medium, and high) to the individual protein antigens between the ASD and TD groups. However, we found several ASD-specific autoantibody reactivity patterns for combinations of two or more antigens (Supplementary Table 2). To identify and validate these patterns of reactivity, we performed ML subgroup discovery analysis, evaluating all possible combinations containing at least of three of the eight antigens (CRMP1, CRMP2, GDA, LDHA, LDHB, NSE, STIP1, and YBOX). We then evaluated the association of a particular pattern with ASD by Fisher’s Exact Test of Independence. Supplementary Table 5 shows the performance of the ASD-specific patterns found in the training set when tested on the validation set.

### Pattern discovery

**Fig. 1:**
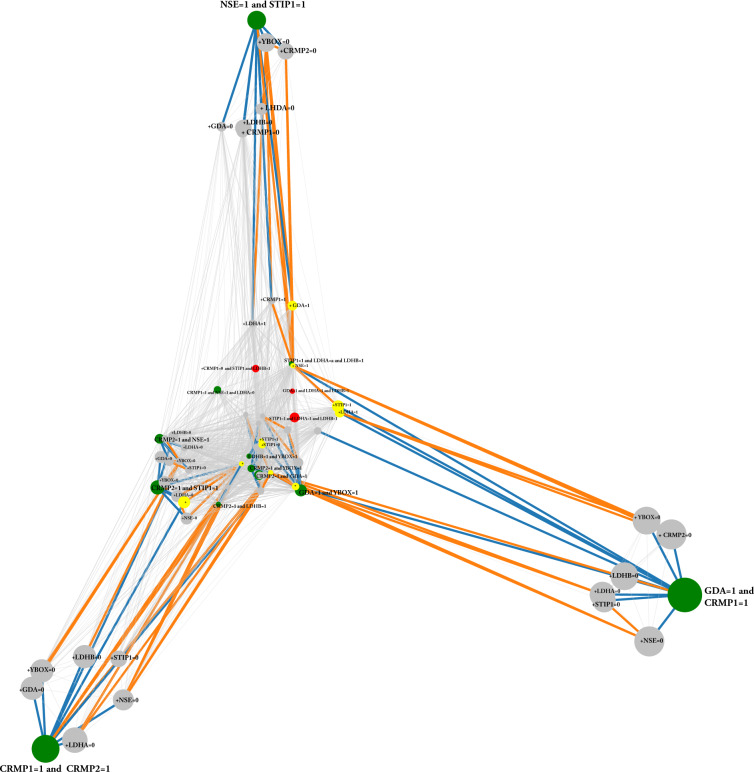
Dependency network of maternal autoantibody reactivity patterns. This network shows the top 70 patterns of autoantibody reactivity predictive of autism spectrum disorder. Each node represents a pattern. The closer two nodes are, the more similar are the sets of samples they cover. The bigger a node and the label, the more samples are covered by the corresponding pattern. Patterns identified by green nodes and bold fonts have 100% precision in the training as well as in the validation set; gray patterns are patterns that also have 100% precision in the training and the validation set, but are sub-patterns of at least one a green pattern, i.e., they cover a subset of samples of the green parent pattern (given this relation, they may be considered redundant); red patterns are patterns that are perfect with regard to precision in the training set but some samples fail in the validation set. Yellow patterns are also 100% precise in the training set but are absent in the validation set. Orange connections mean that the set of samples the bigger of one of the connected patterns covers is a subset of the set of samples the smaller connected pattern covers, while blue connections mean that the description of one pattern is a subset of another pattern (note that blue implies orange). CRMP1 and CRMP2 collapsin response mediator 1 and 2, GDA guanine deaminase, NSE neuron-specific enolase, LDHA-B lactate dehydrogenase A and B, STIP1 stress-induced phosphoprotein 1, and YBOX Y-box binding protein 1. We see that ASD is identified by three major patterns namely “CRMP1 = 1 AND CRMP2 = 1”, “STP = 1 AND NSE = 1” as well as “CRMP = 1 AND GDA = 1”. Most of the other patterns are sub-patterns or represent only a very small set of covered samples.

The patterns of reactivity discovered via subgroup analysis are tightly interconnected in their description as well as in what samples they cover, shown in Fig. 1. These patterns of reactivity were used to build a high precision model to predict ASD outcome (quality analysis; Supplementary Fig. 1). Each node represents a pattern; nodes that are close together cover similar sample subpopulations and the node size is directly proportional to the number of samples covered.

Green nodes represent patterns that have 100% precision in the training and in the validation set, and are the most abundant (CRMP1 + CRMP2, CRMP1 + GDA, and NSE + STIP1). Gray nodes are patterns that also have 100% precision in the training and the validation sets and are sub-patterns of the green patterns. Red and yellow nodes are perfect patterns (patterns that are only found in the ASD population) in the training set, but some fail in the validation set (red) or are absent in validation set (yellow). These patterns are highly interconnected, and orange connections mean that the set of samples the smaller of the connected pattern covers is a subset of the set of samples the bigger pattern covers. Blue connections mean that the connected pattern is a subset of another pattern. The latter (description relation) implies the former (instance relation). We see that three prominent pattern groups emerge at the outer areas of the network, with nonoverlapping sample populations, while the other patterns are mostly specializations (sub-patterns) of these main antigen combinations. Table 1 shows the most representative patterns detected by subgroup discovery, with GDA + CRMP1, CRMP1 + CRMP2, and STIP1 + NSE (and sub-patterns containing reactivity to these antigens) as the main combinations associated with ASD outcome. These patterns cover up to 18% of the ASD cases with 100% precision in the training set, and up to 10% of the ASD cases in the validation data.

**Table 1:** Summary of most relevant autoantibody-antigen reactivity combinations that are 100% specific with an ASD diagnosis in training and validation sets.

Patterns	ASD + training set *n* (%)	TD + training set *n* (%)	Fisher exact *p* value	ASD + validation set *n* (%)	TD + validation set *n* (%)	Training/validation precision (%)	Fisher exact *p* value
CRMP1 + GDA	15 (7)	0 (0)	**0.0002**	4 (2)	0 (0)	100	0.1443
CRMP1 + CRMP2	12 (6)	0 (0)	**0.0007**	4 (2)	0 (0)	100	0.1443
NSE + STIP1	8 (4)	0 (0)	**0.0093**	6 (2)	0 (0)	100	**0.0435**
CRMP2 + STIP1	6 (3)	0 (0)	**0.0346**	3 (1)	0 (0)	100	0.2693
GDA + YBOX	5 (2)	0 (0)	0.0669	1 (0)	0 (0)	100	1
CRMP2 + GDA	4 (2)	0 (0)	0.1303	1 (0)	0 (0)	100	1
CRMP2 + NSE	4 (2)	0 (0)	0.1303	3 (1)	0 (0)	100	0.2693
LDHA + NSE + STIP1	3 (1)	0 (0)	0.2553	1 (0)	0 (0)	100	1
CRMP1 + GDA + LDHB	3 (1)	0 (0)	0.2553	1 (0)	0 (0)	100	1
CRMP2 + YBOX	3 (1)	0 (0)	0.2553	2 (1)	0 (0)	100	0.5129
CRMP1 + NSE + STIP1	3 (1)	0 (0)	0.2553	2 (1)	0 (0)	100	0.5129
CRMP1 + CRMP2 + NSE	3 (1)	0 (0)	0.2553	1 (0)	0 (0)	100	1

Fisher’s exact test (two sided) was used to evaluate the association of the patterns with ASD diagnosis and *p* values >0.05 were bolded and considered significant. The training set was composed of 375 samples (ASD = 206, TD = 169) and the validation set was composed of 418 samples (ASD = 244, TD = 174).

*ASD* autism spectrum disorders, *TD* typically developing, *CRMP1 and CRMP2* collapsin response mediator proteins 1 and 2, *GDA* guanine deaminase, *NSE* neuron-specific enolase, *LDHA-B* lactate dehydrogenase A and B, *STIP1* stress-induced phosphoprotein 1, and *YBOX* Y-box binding protein 1.

Using the training set, we identified 12 autoantibody pattern combinations that were considered ASD-relevant based on having 3 or more positive ASD and no TD subjects with a particular pattern of reactivity and were also present in the validation set (Table 1). CRMP1 + GDA (*n* = 15, *p* = 0.0002), CRMP1 + CRMP2 (*n* = 12, *p* = 0.0007), NSE + STIP1 (*n* = 8, *p* = 0.0093), and CRMP2 + STIP1 (*n* = 6, *p* = 0.0346) were the most highly represented combinations with the greatest statistical significance. We then used the validation sample set to evaluate pattern accuracy for the prediction of an ASD outcome.

In the validation set, the most abundant pattern was NSE + STIP1 (*n* = 6, *p* = 0.0435), followed by CRMP1 + GDA and CRMP1 + CRMP2 (both *n* = 4, *p* = 0.1443), CRMP2 + STIP1 and CRMP2 + NSE (both *n* = 3, *p* = 0.2693) that were ASD-specific patterns in training and validation sets but did not reach statistical significance. Of interest, we identified STIP1 + YBOX as an ASD-specific pattern in the validation set (*n* = 7, *p* = 0.0448), however, that pattern was not ASD-specific in the training set (4 ASD vs. 1 TD), resulting in 92% of samples having an ASD diagnosis when considering both sample sets (Table 2 and Supplementary Tables 5, 6).

**Table 2:** Summary of clinically-relevant statistics of autoantibody-antigen reactivity combinations that are from 100 to 90% specific with ASD diagnosis in the training and validation set (combined data).

Pattern	Training ASD + /subgroup	Validation ASD + /subgroup	ASD/subgroup	Precision	Precision drop	Total ASD % (*n* = 450)	Fisher’s exact test *p* value	OR (95% CI)^a^	OR *p* value
CRMP1 + GDA	15/15	4/4	19/19	1.00	0.00	4.2	**<0.0001**	**31.04 (1.8678–516.0620)**	0.0166
CRMP1 + CRMP2	12/12	4/4	16/16	1.00	0.00	3.6	**0.0001**	**26.08 (1.5596–436.4170)**	0.0233
NSE + STIP1	8/8	6/6	14/14	1.00	0.00	3.1	**0.0005**	**22.82 (1.3565–383.9379)**	0.0299
CRMP2 + STIP1	6/6	3/3	9/9	1.00	0.00	2	**0.0064**	**14.78 (0.8573–254.8841)**	0.0637
LDHA + YBOX	1/1	5/5	6/6	1.00	0.00	1.3	**0.0393**	**10.04 (0.5640–178.9532)**	0.1164
LDHB + YBOX	2/2	4/4	6/6	1.00	0.00	1.3	**0.0393**	**10.04 (0.5640–178.9532)**	0.1164
GDA + YBOX	5/5	1/1	6/6	1.00	0.00	1.3	**0.0393**	**10.04 (0.5640–178.9532)**	0.1164
STIP1 + YBOX	4/5	7/7	11/12	*0.92*	*0.08*	*2.4*	***0.0161***	***8.56 (1.1010–66.7020)***	0.0402
CRMP1 + STIP1	15/17	3/3	18/20	*0.90*	*0.10*	*4*	***0.0022***	***7.10 (1.6371–30.8292)***	0.0088

Fisher’s exact (two sided) was used to evaluate the association of the patterns with ASD diagnosis and *p* values >0.05 were bolded and considered significant. The italic values represent significant combinations that are not 100% ASD-specific, but are statistically significant and have a strong correlation with the ASD group.

^a^ A 0.5 continuity correction was applied to OR calculations for observations with zero cell counts. The correction was applied to all OR calculations in this table, except the last two patterns (STIP1+YBOX and CRMP1+STIP1).

*ASD* autism spectrum disorders, *OR* odds ratio, *CI* confidence interval, *CRMP1 and CRMP2* collapsin response mediator proteins 1 and 2, *GDA* guanine deaminase, *NSE* neuron-specific enolase, *LDHA-B* lactate dehydrogenase A and B, *STIP1* stress-induced phosphoprotein 1, and *YBOX* Y-box binding protein 1.

Table 2 presents a summary of clinically-relevant statistics of autoantibody–antigen reactivity combinations that are 90–100% specific with ASD diagnosis in the training and validation sets. In order to evaluate the association of a given pattern with ASD, we used the Fisher Exact Test and calculated the odds ratios (ORs) with 95% confidence intervals (95% CIs) for each primary pattern (including the sub-patterns) from the entire sample set (ASD = 450, TD = 343). The ASD-specific combinations that had odd ratios ≥10 and were statistically significant (*p* = 0.05) included CRMP1 + GDA (OR 31.04, 95% CI: 1.8678–516.0620, *p* < 0.0001), CRMP1 + CRMP2 (OR 26.08, 95% CI: 1.5596–436.4170, *p* = 0.0005), NSE + STIP1 (OR 22.82, 95% CI: 1.3565–383.9379, *p* = 0.0001) and CRMP2 + STIP1 (OR 14.78, C95% CI: 0.8573–254.8841, *p* = 0.0064). We found two patterns that were not 100% specific for ASD: STIP1 + YBOX (ASD = 11 vs. TD = 1, *p* = 0.0161) and CRMP1 + STIP1 (ASD = 18, TD = 2, *p* = 0.0022), with an ASD prediction accuracy of 92% and 90% respectively. The association of these two patterns with ASD was statistically significant in both cases.

### ADOS correlation

To study the relationship between autoantibody reactivity against the eight antigens with the ADOS severity score, we evaluated the 254 ASD samples that were positive for any given antigen using stepwise and LASSO selection models and calculated the odds ratio and 95% confidence interval. Out of the eight antigens, CRMP1 had the strongest correlation with ADOS severity, with an odds ratio of 2.3 (95% CI: 1. 358–3.987, *p* = 0.0021), meaning that having autoantibodies against CRMP1 increases the risk of having a more severe overall ADOS score by 2.3 (Table 3).

**Table 3:** Ordinal logistic regression for ADOS severity.

Variable	Point estimate	95% confidence interval	*p* value
CRMP1	**2.327**	**1.358–3.987**	**0.0021**
CRMP2	0.589	0.284–1.22	0.1541
GDA	0.702	0.421–1.172	0.1764
LDHA	1.011	0.601–1.702	0.9676
LDHB	0.947	0.544–1.649	0.8484
NSE	1.106	0.598–2.046	0.7479
STP1	0.927	0.575–1.493	0.7544
YBOX	0.858	0.451–1.63	0.6391

Stepwise and LASSO selection models were used to calculate the odds ratio and 95% confidence interval and *p* values >0.05 were bolded and considered significant.

*ADOS* autism diagnostic observation schedule, *CRMP1 and CRMP2* collapsin response mediator proteins 1 and 2, *GDA* guanine deaminase, *NSE* neuron-specific enolase, *LDHA-B* lactate dehydrogenase A and B, *STIP1* stress-induced phosphoprotein 1, and *YBOX* Y-box binding protein 1, *LASSO* least absolute shrinkage and selection operator.

## Discussion

Several groups have shown that the presence of deleterious maternal autoantibodies against fetal brain proteins can result in permanent neurodevelopmental and behavioral alterations in the progeny [15–23]. The mechanisms and dynamics of how the maternal antibodies are able to cross the fetal blood brain barrier, transfer to the fetal brain parenchyma where are taken up by the neural progenitor cells to bind the intracellular targets is still unknown. Further, it has been proposed that autoantibodies against brain antigens can act as agonistic, antagonist or co-agonist antibodies on surface receptors, altering receptor signaling, fix complement, and/or activating Fc surface receptors (cell death) [24].

To address the potential pathogenicity of the MAR autoantibodies, we previously created several animal models, both passive transfer models using human IgG reactive to the antigens [25–28], as well as the creation of an endogenous mouse model in which we generated clinically-relevant autoantibodies in the dam prior to breeding [29]. In our MAR rodent models, we have not observed tissue damage histologically, but we have found that maternal autoantibodies affect progenitor cell maturation resulting in altered dendritic maturation. For example, in Martínez-Cerdeño et al. when biotin-labeled human ASD-specific IgG antibodies to LDHA, LDHB, STIP1, and CRMP1 were injected into the mouse cerebral ventricles at embryonic day 14.5, we noted specific intracellular autoantibody deposition in radial glial stem cells, and further noted abnormal radial glial cell proliferation, maturation, and alteration of mature dendritic structure [30, 31]. These findings demonstrate that the maternal IgG antibodies can bind to their intracellular targets in vivo. The mechanism of this uptake by the proliferating radial glial cells is currently under investigation.

In the endogenous mouse model, the developing pups were exposed throughout gestation to pathogenic antibodies against LDHA, LDHB, STIP1, and CRMP1. Exposed pups showed ASD-like behavioral alterations, including reduced vocalizations, increased repetitive self-grooming, and aberrant social interactions [29], demonstrating for the first time the true pathological significance of these autoantibodies.

We have reported in each of our studies that reactivity to an individual autoantigen is present to some degree in both groups (ASD and TD) and does not correlate with an ASD diagnosis [4]. Instead, reactivity to a combination of two or more autoantigens is necessary to determine an association of risk for ASD. This phenomenon, where detection of more than one autoantibody its necessary to accurately predict disease risk, has been reported for other autoimmune diseases, such as Type 1 diabetes [32].

Other groups have searched for individual IgG-targeted autoantigens that could serve as a biomarker for ASD. Lee et al. demonstrated the neurotoxic effects of gestational exposure to monoclonal anti-NMDAR (N-methyl-D-aspartate) that resulted in morphological alterations in the developing brain causing long-term cognitive effects in the exposed pups. However, these offspring did not exhibit the specific behavioral changes related to ASD [23]. Maternal antibodies to contactin-associated protein-like 2 (CASPR2) have been reported to be associated with neurodevelopmental alterations and behavioral aberrations related to ASD [22]. However, such an association was not observed in a recent Danish study concluding that maternal autoantibodies to CASPR2 were highly associated with child diagnosis of intellectual disability and/or psychological development disorders, but not with ASD [33]. Therefore, antibodies that interfere with NMDAR and CASPR2 function appear to have profound effects in neurodevelopment, causing brain abnormalities (observed in murine models) and permanent behavioral aberrations, yet additional studies are necessary to evaluate their utility as ASD biomarkers [21, 23, 34].

**Fig. 2:**
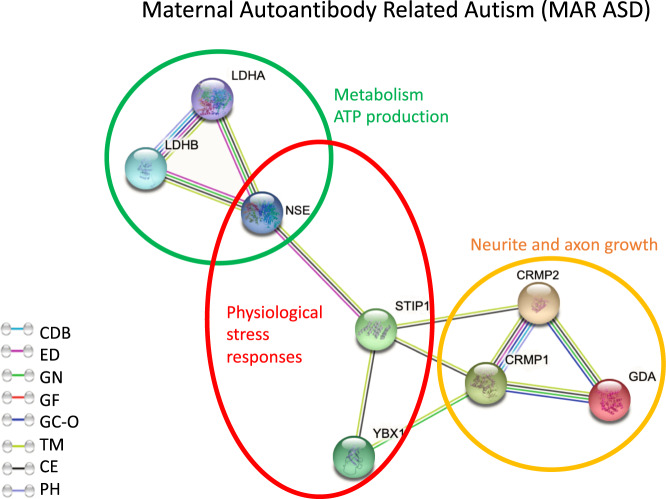
STRING protein-protein interaction network of the known MAR-ASD antigens. Each antigen is presented as a network node, and the edges represent meaningful protein-protein associations. CDB curated data bases, ED experimentally determined, GN gene neighborhood, GF gene fusions, GC-O gene co-occurrence, TM text mining, CE co-expression, PH protein homology.

The primary goal of the current study was to build upon our previous findings to develop an optimized, quantitative ELISA assay able to detect the presence of maternal autoantibodies specific to ASD risk using ML tools. We found that the most common MAR-ASD patterns were CRMP1 + CRMP2, CRMP1 + GDA, NSE + STIP1, and GDA + YBOX. These patterns were 100% accurate for the prediction of ASD in both the training and validation sets, suggesting that autoantibodies to these antigen combinations are highly related to an ASD diagnosis and have the potential to be used as biomarkers of MAR-ASD risk. As illustrated in Fig. 2, the autoantigens are highly interconnected by their tissue expression, biological function, and/or structural similarities as represented in the STRING network [35]. The target proteins are highly expressed in the developing brain, and play important roles in neurogenesis, metabolism, and homeostasis [15, 16, 18]. Therefore, it is possible that antibody binding during this vulnerable period could impact proper protein functionality affecting significant neurodevelopmental pathways with a lasting effect on the developing brain.

One of the primary MAR-ASD-specific patterns, CRMP1 and CRMP2 are involved in axon and neurite guidance in the nervous system [36–38]. Knockout mice (CRMP1^−/−^ and CRMP2^−/−^) present behavioral deficits including hyperactivity, increased locomotion, and deficits in social behavior and memory [39]. CRMP1/2 expression and phosphorylation has been proposed as a biomarker for several neuropsychiatric pathologies including Alzheimer disease, schizophrenia, and epilepsy [36]. GDA (Cypin) has important catabolic and structural functions in neurons, thus regulating dendrite patterning and synaptic development and plasticity [40]. Therefore, autoantibodies to GDA + CRMP1—the most abundant pattern found in the current study—could interfere with two independent neurite and axon development pathways, which could have serious implications during neurodevelopment.

STIP1 modulates several biological process including physiological stress responses, signal transduction, transcription, and cell cycle regulation [41]. These observations are complemented by animal studies where STIP1^+/−^ mice (lower expression of STIP1) showed ASD-like behaviors including attention deficits and hyperactivity [42]. These findings suggest that reduced expression or functionality of STIP1 could be used as a biomarker for ASD. NSE is an enolase enzyme with glycolytic activity involved in ATP, and has been shown to mediate the PI3K activation pathway, having neuroprotective or neurogenerative effects depending on the strength of the signal [43]. As noted, NSE + STIP1 is the third most abundant ASD-specific pattern, and both proteins have been described to play important roles in neurodevelopment, brain homeostasis, and especially neuroprotection under physiologic stress conditions. Thus, interference in the function of both STIP1 and NSE could result in neurodevelopmental abnormalities and an insufficient response to cellular stress.

It was previously reported that maternal autoantibody reactivity to fetal brain antigens correlated with distinct ASD manifestations in the affected children [8] including increased irritability and language deficits. One intriguing finding in the current study was the association between autoantibodies against CRMP1 and worse ASD manifestations based on the ADOS severity score. While additional studies are underway to better understand the endophenotypes within MAR-ASD as they relate to the various MAR patterns, our current findings serve as a strong foundation to further examine these interactions using a larger data set as well as looking at other ASD metrics and subcategories.

Although this is the largest study to date in terms of sample size for MAR-ASD, we are still limited in our ability to significantly detect ASD patterns with lower frequencies. Additionally, this is a retrospective study, as the samples were collected ~2–3 years after delivery and at the time of the child’s diagnosis. Recognizing the importance of a prospective analysis, we are currently evaluating the MAR-ASD patterns described herein in additional prospective studies as well as in geographically distinct study populations. Likewise, new animal models are underway to evaluate the patterns of reactivity described in the current clinical population allowing assessment of the pathogenic effect of antibodies against individual or specific combinations of antigens.

In conclusion, this is the first report that uses ML to identify a set of biomarkers that demonstrate an association with MAR-ASD with 100% accuracy. This is a novel serological risk assessment test for women at high risk of having a child with ASD; for example, those mothers that have previously had a child diagnosed on the spectrum or that have other ASD-associated maternal co-morbidities such as metabolic syndrome during pregnancy [44]. While the use of this technology in the clinical population will require substantial clinical validation and testing, this study provides a strong foundation for such studies in the future and provides a framework for understanding the biologic implications of MAR-autoantibodies in future animal models.

## Supplementary information

Supplementary Figure 1

Supplementary Tables
