# Supplementary material for: Risk assessment analysis for maternal autoantibody-related autism (MAR-ASD): a subtype of autism
Source: Mol Psychiatry. 2021 Jan 22;26(5):1551–60. doi: 10.1038/s41380-020-00998-8 (PMC8159732; doi:10.1038/s41380-020-00998-8)
Supplement: Supplementary file 1 — Supplementary Figure 1 [file 41380_2020_998_MOESM1_ESM.pdf]

**Supplementary Figure 1. Performance of pattern-based prediction.**

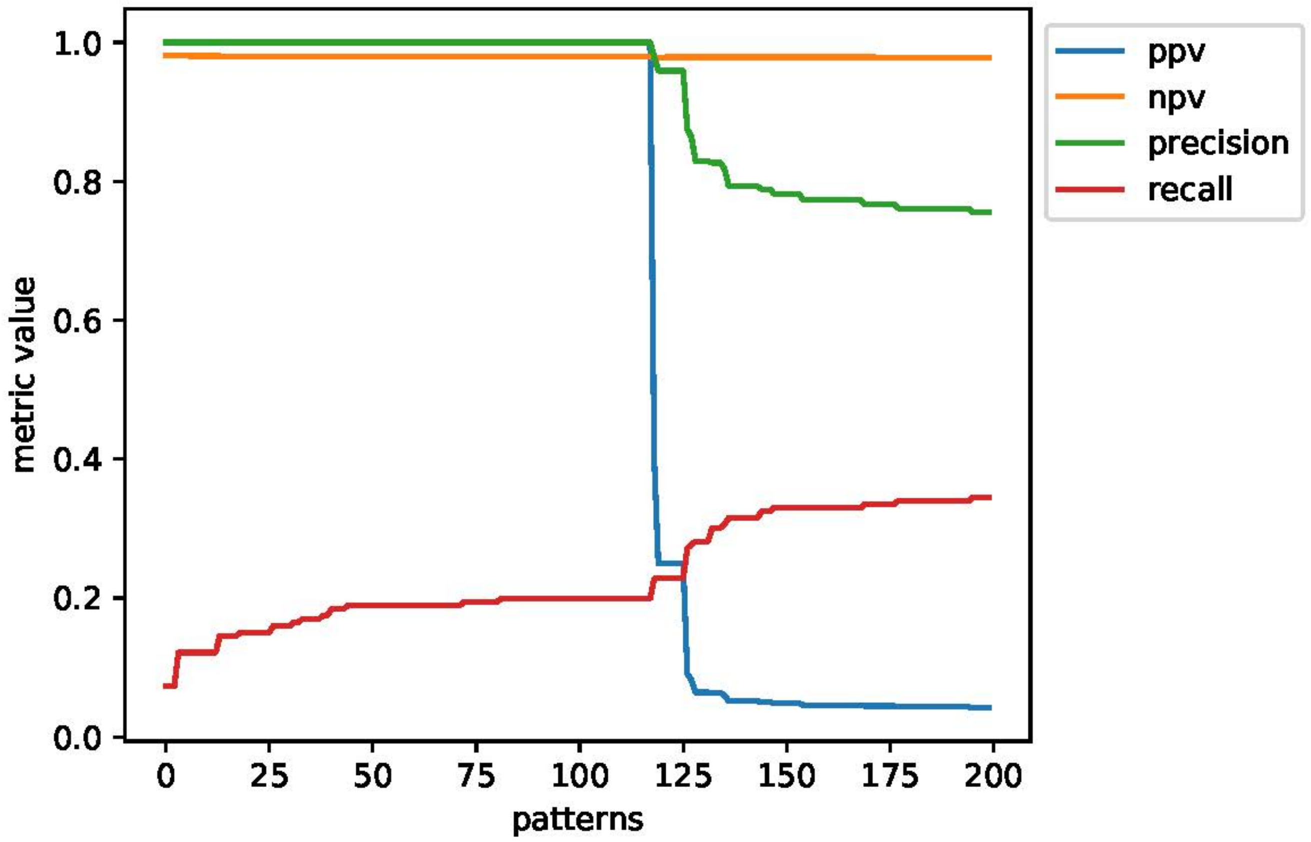


Supplementary Figure 1. This figure illustrates the positive predictive value (PPV), negative predictive value (NPV), precision and recall of the pattern-based predictor. A) depicts the performance on the training set, while B) demonstrates the performance on the validation dataset. The x-axis shows the influence of the number of top-k patterns used for prediction.
