# Supplementary material for: Risk assessment analysis for maternal autoantibody-related autism (MAR-ASD): a subtype of autism
Source: Mol Psychiatry. 2021 Jan 22;26(5):1551–60. doi: 10.1038/s41380-020-00998-8 (PMC8159732; doi:10.1038/s41380-020-00998-8)
Supplement: Supplementary file 2 — Supplementary Tables [file 41380_2020_998_MOESM2_ESM.docx]

**Supplementary Table 1. Demographics of study population. Illustrates the mean maternal age at birth of child and mean age at time of sample collection.**

| Diagnosis | ASD | TD |
| --- | --- | --- |
| **Number of subjects^a^ (n)** | 450 | 343 |
| **Mean Maternal age at birth of child (years)** | 30 | 30 |
| **SD** | 6 | 6 |
| **Max Age** | 45 | 44 |
| **Min Age** | 16 | 17 |

Supplementary Table 1. ^a^Subjects from Childhood Autism Risk from Genetics and the Environment (CHARGE) study. Descriptive statistics are shown.

Abbreviations: ASD, Autism Spectrum Disorders; TD, Typically Developing, SD, Standard Deviation, Max, Maximum age, Min, Minimum age.

**Supplementary Table 2. Summary of relevant autoantibody-antigen reactivity combinations that are 100% specific with ASD diagnosis in training and validation sets.**

| ***100% ASD SPECIFIC PATTERNS*** | | | | | | |
| --- | --- | --- | --- | --- | --- | --- |
|  | ASD + Training | ASD + Validation | ASD total | ASD % | Total ASD+/ Subgroup | Precision |
| ***CRMP1 + GDA PATTERNS*** |  |  |  |  |  |  |
| CRMP1 + GDA | 6 | 3 | 9 | 2 | 9/9 | 1.00 |
| CRMP1 + GDA + LDHA | 2 | 0 | 2 | 0.4 | 2/2 | 1.00 |
| CRMP1 + GDA + LDHB | 0 | 1 | 1 | 0.2 | 1/1 | 1.00 |
| CRMP1 + GDA + STIP1 | 1 | 0 | 1 | 0.2 | 1/1 | 1.00 |
| CRMP1 + GDA + YBOX | 1 | 0 | 1 | 0.2 | 1/1 | 1.00 |
| CRMP1+ CRMP2 + GDA + YBOX | 1 | 0 | 1 | 0.2 | 1/1 | 1.00 |
| CRMP1 + GDA + NSE + STIP1 | 1 | 0 | 1 | 0.2 | 1/1 | 1.00 |
| CRMP1 + GDA + LDHA + LDHB + STIP1 | 1 | 0 | 1 | 0.2 | 1/1 | 1.00 |
| CRMP1 + GDA + LDHA + LDHB + NSE +STIP1 | 1 | 0 | 1 | 0.2 | 1/1 | 1.00 |
| CRMP1 + CRMP2 + GDA + LDHB + STIP1 + YBOX | 1 | 0 | 1 | 0.2 | 1/1 | 1.00 |
| **Total** | **15** | **4** | **19** | **4.2** | **19/19** | **1.00** |
|  |  |  |  |  |  |  |
| ***CRMP1 + CRMP2 PATTERNS*** |  |  |  |  |  |  |
| CRMP1 +CRMP2 | 4 | 1 | 5 | 1.1 | 5/5 | 1.00 |
| CRMP1 + CRMP2 + LDHB | 0 | 1 | 1 | 0.2 | 1/1 | 1.00 |
| CRMP1 + CRMP2 + NSE | 2 | 1 | 3 | 0.7 | 3/3 | 1.00 |
| CRMP1 + CRMP2 + STIP1 | 2 | 0 | 2 | 0.4 | 2/2 | 1.00 |
| CRMP1+ CRMP2 + GDA + YBOX | 1 | 0 | 1 | 0.2 | 1/1 | 1.00 |
| CRMP1+ CRMP2 + LDHB + STIP1 | 1 | 0 | 1 | 0.2 | 1/1 | 1.00 |
| CRMP1 + CRMP2 + LDHA + NSE + STIP1 | 1 | 0 | 1 | 0.2 | 1/1 | 1.00 |
| CRMP1 + CRMP2 + LDHA + LDHB + YBOX | 0 | 1 | 1 | 0.2 | 1/1 | 1.00 |
| CRMP1 + CRMP2 + GDA + LDHB + STIP1 + YBOX | 1 | 0 | 1 | 0.2 | 1/1 | 1.00 |
| **Total** | **12** | **4** | **16** | **3.6** | **16/16** | **1.00** |
|  |  |  |  |  |  |  |
| ***NSE + STIP1 PATTERNS*** |  |  |  |  |  |  |
| NSE + STIP1 | 3 | 2 | 5 | 1.1 | 5/5 | 1.00 |
| GDA + NSE + STIP1 | 1 | 0 | 1 | 0.2 | 1/1 | 1.00 |
| CRMP1 + LDHB + NSE + STIP1 | 0 | 2 | 2 | 0.4 | 2/2 | 1.00 |
| CRMP1 + GDA + NSE + STIP1 | 1 | 0 | 1 | 0.2 | 1/1 | 1.00 |
| CRMP2 + NSE + STIP1 + YBOX | 0 | 1 | 1 | 0.2 | 1/1 | 1.00 |
| GDA + LDHA + NSE + STIP1 | 1 | 0 | 1 | 0.2 | 1/1 | 1.00 |
| LDHA + LDHB + NSE + STIP1 | 0 | 1 | 1 | 0.2 | 1/1 | 1.00 |
| CRMP1 + CRMP2 + LDHA + NSE + STIP1 | 1 | 0 | 1 | 0.2 | 1/1 | 1.00 |
| CRMP1 + GDA + LDHA + LDHB + NSE +STIP1 | 1 | 0 | 1 | 0.2 | 1/1 | 1.00 |
| **Total** | **8** | **6** | **14** | **3.1** | **14/14** | **1.00** |
|  |  |  |  |  |  |  |
| ***LDHA + YBOX PATTERNS*** |  |  |  |  |  |  |
| LDHA + YBOX | 1 | 3 | 4 | 0.9 | 4/4 | 1.00 |
| LDHA + STIP1 + YBOX | 0 | 1 | 1 | 0.2 | 1/1 | 1.00 |
| CRMP1 + CRMP2 + LDHA + LDHB + YBOX | 0 | 1 | 1 | 0.2 | 1/1 | 1.00 |
| **Total** | **1** | **5** | **6** | **1.3** | **6/6** | **1.00** |
|  |  |  |  |  |  |  |
| ***LDHB + YBOX PATTERNS*** |  |  |  |  |  |  |
| LDHB + YBOX | 1 | 0 | 1 | 0.2 | 1/1 | 1.00 |
| LDHB + NSE + YBOX | 1 | 1 | 2 | 0.4 | 2/2 | 1.00 |
| LDHB + STIP1+ YBOX | 0 | 1 | 1 | 0.2 | 1/1 | 1.00 |
| CRMP1 + CRMP2 + LDHA + LDHB + YBOX | 0 | 1 | 1 | 0.2 | 1/1 | 1.00 |
| CRMP1 + CRMP2 + GDA + LDHB + STIP1 + YBOX | 1 | 0 | 1 | 0.2 | 1/1 | 1.00 |
| **Total** | **3** | **3** | **6** | **1.3** | **6/6** | **1.00** |
|  |  |  |  |  |  |  |
| ***CRMP2 + STIP1 PATTERNS*** |  |  |  |  |  |  |
| CRMP2 + STIP1 | 1 | 1 | 2 | 0.4 | 2/2 | 1.00 |
| CRMP2 + GDA + STIP1 | 0 | 1 | 1 | 0.2 | 1/1 | 1.00 |
| CRMP1 + CRMP2 + STIP1 | 2 | 0 | 2 | 0.4 | 2/2 | 1.00 |
| CRMP2 + NSE + STIP1 + YBOX | 0 | 1 | 1 | 0.2 | 1/1 | 1.00 |
| CRMP1+ CRMP2 + LDHB + STIP1 | 1 | 0 | 1 | 0.2 | 1/1 | 1.00 |
| CRMP1 + CRMP2 + LDHA + NSE + STIP1 | 1 | 0 | 1 | 0.2 | 1/1 | 1.00 |
| CRMP1 + CRMP2 + GDA + LDHB + STIP1 + YBOX | 1 | 0 | 1 | 0.2 | 1/1 | 1.00 |
| **Total** | **6** | **3** | **9** | **2.0** | **8/8** | **1.00** |
|  |  |  |  |  |  |  |
| ***GDA + YBOX PATTERNS*** |  |  |  |  |  |  |
| GDA + YBOX | 1 | 1 | 2 | 0.4 | 2/2 | 1.00 |
| GDA + STIP1 + YBOX | 1 | 0 | 1 | 0.2 | 1/1 | 1.00 |
| CRMP1 + GDA + YBOX | 1 | 0 | 1 | 0.2 | 1/1 | 1.00 |
| CRMP1+ CRMP2 + GDA + YBOX | 1 | 0 | 1 | 0.2 | 1/1 | 1.00 |
| CRMP1 + CRMP2 + GDA + LDHB + STIP1 + YBOX | 1 | 0 | 1 | 0.2 | 1/1 | 1.00 |
| **Total** | **5** | **1** | **6** | **1.3** | **6/6** | **1.00** |
|  |  |  |  |  |  |  |

Supplementary Table 2. Abbreviations: ASD, Autism Spectrum Disorders; TD, Typically Developing. CRMP1 and CRMP2, collapsin response mediator proteins 1 and 2; GDA, guanine deaminase; NSE, neuron specific enolase; LDHA-B, lactate dehydrogenase A and B; STIP1, stress induced phosphoprotein 1 and YBOX, Y-box binding protein 1.

**Supplementary Table 3. Illustrates the top 70 patterns identified by machine learning.**

| **Pattern** | **Training: ASD + /subgroup size** | **Training: precision** | **Validation: ASD + / subgroup size** | **Validation: precision** | **Precision drop** |
| --- | --- | --- | --- | --- | --- |
| crmp1=1 AND gda=1 | 15 / 15 | 1.00 | 4 / 4 | 1.00 | 0.00 |
| crmp1=1 AND gda=1 AND nse=0 | 13 / 13 | 1.00 | 4 / 4 | 1.00 | 0.00 |
| crmp1=1 AND crmp2=0 AND gda=1 | 13 / 13 | 1.00 | 4 / 4 | 1.00 | 0.00 |
| crmp1=1 AND crmp2=1 | 12 / 12 | 1.00 | 4 / 4 | 1.00 | 0.00 |
| crmp1=1 AND gda=1 AND ldhb=0 | 12 / 12 | 1.00 | 3 / 3 | 1.00 | 0.00 |
| crmp1=1 AND gda=1 AND ybox=0 | 12 / 12 | 1.00 | 4 / 4 | 1.00 | 0.00 |
| crmp1=1 AND crmp2=1 AND ldha=0 | 11 / 11 | 1.00 | 3 / 3 | 1.00 | 0.00 |
| crmp1=1 AND gda=1 AND ldha=0 | 11 / 11 | 1.00 | 4 / 4 | 1.00 | 0.00 |
| crmp1=1 AND crmp2=1 AND gda=0 | 10 / 10 | 1.00 | 4 / 4 | 1.00 | 0.00 |
| crmp1=1 AND crmp2=1 AND ldhb=0 | 10 / 10 | 1.00 | 2 / 2 | 1.00 | 0.00 |
| crmp1=1 AND crmp2=1 AND ybox=0 | 10 / 10 | 1.00 | 3 / 3 | 1.00 | 0.00 |
| crmp1=1 AND gda=1 AND stp1=0 | 10 / 10 | 1.00 | 4 / 4 | 1.00 | 0.00 |
| crmp1=1 AND crmp2=1 AND nse=0 | 9 / 9 | 1.00 | 3 / 3 | 1.00 | 0.00 |
| nse=1 AND stp1=1 AND ybox=0 | 8 / 8 | 1.00 | 5 / 5 | 1.00 | 0.00 |
| nse=1 AND stp1=1 | 8 / 8 | 1.00 | 6 / 6 | 1.00 | 0.00 |
| crmp1=1 AND crmp2=1 AND stp1=0 | 7 / 7 | 1.00 | 4 / 4 | 1.00 | 0.00 |
| ldhb=0 AND nse=1 AND stp1=1 | 7 / 7 | 1.00 | 3 / 3 | 1.00 | 0.00 |
| crmp2=0 AND nse=1 AND stp1=1 | 7 / 7 | 1.00 | 5 / 5 | 1.00 | 0.00 |
| crmp2=1 AND stp1=1 | 6 / 6 | 1.00 | 3 / 3 | 1.00 | 0.00 |
| crmp1=0 AND nse=1 AND stp1=1 | 5 / 5 | 1.00 | 4 / 4 | 1.00 | 0.00 |
| crmp2=1 AND gda=0 AND stp1=1 | 5 / 5 | 1.00 | 2 / 2 | 1.00 | 0.00 |
| crmp2=1 AND ldha=0 AND stp1=1 | 5 / 5 | 1.00 | 3 / 3 | 1.00 | 0.00 |
| crmp2=1 AND nse=0 AND stp1=1 | 5 / 5 | 1.00 | 2 / 2 | 1.00 | 0.00 |
| crmp2=1 AND stp1=1 AND ybox=0 | 5 / 5 | 1.00 | 2 / 2 | 1.00 | 0.00 |
| crmp1=1 AND crmp2=1 AND stp1=1 | 5 / 5 | 1.00 | -1 / -1 | -1.00 | -1.00 |
| ldha=0 AND nse=1 AND stp1=1 | 5 / 5 | 1.00 | 5 / 5 | 1.00 | 0.00 |
| gda=1 AND ldha=0 AND ybox=1 | 5 / 5 | 1.00 | 1 / 1 | 1.00 | 0.00 |
| gda=1 AND nse=0 AND ybox=1 | 5 / 5 | 1.00 | 1 / 1 | 1.00 | 0.00 |
| crmp1=1 AND gda=1 AND stp1=1 | 5 / 5 | 1.00 | -1 / -1 | -1.00 | -1.00 |
| gda=1 AND ybox=1 | 5 / 5 | 1.00 | 1 / 1 | 1.00 | 0.00 |
| crmp2=1 AND ldhb=0 AND stp1=1 | 4 / 4 | 1.00 | 3 / 3 | 1.00 | 0.00 |
| crmp2=1 AND ldhb=0 AND nse=1 | 4 / 4 | 1.00 | 3 / 3 | 1.00 | 0.00 |
| crmp2=1 AND nse=1 AND ybox=0 | 4 / 4 | 1.00 | 2 / 2 | 1.00 | 0.00 |
| crmp2=1 AND gda=1 | 4 / 4 | 1.00 | 1 / 1 | 1.00 | 0.00 |
| crmp2=1 AND nse=1 | 4 / 4 | 1.00 | 3 / 3 | 1.00 | 0.00 |
| gda=0 AND nse=1 AND stp1=1 | 4 / 4 | 1.00 | 6 / 6 | 1.00 | 0.00 |
| gda=1 AND ldhb=0 AND ybox=1 | 4 / 4 | 1.00 | 1 / 1 | 1.00 | 0.00 |
| gda=1 AND nse=1 AND stp1=1 | 4 / 4 | 1.00 | -1 / -1 | -1.00 | -1.00 |
| gda=1 AND ldhb=1 AND stp1=1 | 4 / 4 | 1.00 | 0 / 1 | 0.00 | 1.00 |
| crmp1=1 AND gda=1 AND ldha=1 | 4 / 4 | 1.00 | -1 / -1 | -1.00 | -1.00 |
| crmp1=0 AND ldhb=1 AND stp1=1 | 3 / 3 | 1.00 | 6 / 11 | 0.55 | 0.45 |
| crmp2=1 AND gda=0 AND nse=1 | 3 / 3 | 1.00 | 3 / 3 | 1.00 | 0.00 |
| crmp2=1 AND ldha=0 AND nse=1 | 3 / 3 | 1.00 | 2 / 2 | 1.00 | 0.00 |
| crmp2=1 AND gda=1 AND ldha=0 | 3 / 3 | 1.00 | 1 / 1 | 1.00 | 0.00 |
| crmp2=1 AND ldha=0 AND ybox=1 | 3 / 3 | 1.00 | 1 / 1 | 1.00 | 0.00 |
| crmp2=1 AND gda=1 AND ldhb=0 | 3 / 3 | 1.00 | 1 / 1 | 1.00 | 0.00 |
| crmp2=1 AND gda=1 AND nse=0 | 3 / 3 | 1.00 | 1 / 1 | 1.00 | 0.00 |
| crmp2=1 AND nse=0 AND ybox=1 | 3 / 3 | 1.00 | 1 / 1 | 1.00 | 0.00 |
| crmp2=1 AND gda=1 AND stp1=0 | 3 / 3 | 1.00 | -1 / -1 | -1.00 | -1.00 |
| crmp2=1 AND nse=1 AND stp1=0 | 3 / 3 | 1.00 | 2 / 2 | 1.00 | 0.00 |
| crmp1=1 AND crmp2=1 AND nse=1 | 3 / 3 | 1.00 | 1 / 1 | 1.00 | 0.00 |
| crmp2=1 AND ybox=1 | 3 / 3 | 1.00 | 2 / 2 | 1.00 | 0.00 |
| crmp1=1 AND ldha=0 AND nse=1 | 3 / 3 | 1.00 | 3 / 3 | 1.00 | 0.00 |
| crmp1=1 AND nse=1 AND stp1=1 | 3 / 3 | 1.00 | 2 / 2 | 1.00 | 0.00 |
| ldha=1 AND nse=1 AND stp1=1 | 3 / 3 | 1.00 | 1 / 1 | 1.00 | 0.00 |
| gda=1 AND stp1=0 AND ybox=1 | 3 / 3 | 1.00 | 1 / 1 | 1.00 | 0.00 |
| crmp1=1 AND gda=1 AND ldhb=1 | 3 / 3 | 1.00 | 1 / 1 | 1.00 | 0.00 |
| crmp1=1 AND gda=1 AND ybox=1 | 3 / 3 | 1.00 | -1 / -1 | -1.00 | -1.00 |
| crmp2=0 AND gda=1 AND ybox=1 | 3 / 3 | 1.00 | 1 / 1 | 1.00 | 0.00 |
| crmp1=0 AND crmp2=1 AND gda=1 | 2 / 2 | 1.00 | 1 / 1 | 1.00 | 0.00 |
| crmp1=0 AND gda=1 AND ybox=1 | 2 / 2 | 1.00 | 1 / 1 | 1.00 | 0.00 |
| crmp2=1 AND ldha=0 AND ldhb=1 | 2 / 2 | 1.00 | 1 / 1 | 1.00 | 0.00 |
| crmp2=1 AND ldhb=0 AND ybox=1 | 2 / 2 | 1.00 | 1 / 1 | 1.00 | 0.00 |
| crmp2=1 AND ldhb=1 AND nse=0 | 2 / 2 | 1.00 | 2 / 2 | 1.00 | 0.00 |
| crmp2=1 AND ldhb=1 AND stp1=1 | 2 / 2 | 1.00 | -1 / -1 | -1.00 | -1.00 |
| crmp2=1 AND gda=1 AND ybox=0 | 2 / 2 | 1.00 | 1 / 1 | 1.00 | 0.00 |
| crmp1=1 AND crmp2=1 AND gda=1 | 2 / 2 | 1.00 | -1 / -1 | -1.00 | -1.00 |
| crmp2=1 AND gda=1 AND ybox=1 | 2 / 2 | 1.00 | -1 / -1 | -1.00 | -1.00 |
| crmp2=1 AND stp1=0 AND ybox=1 | 2 / 2 | 1.00 | 1 / 1 | 1.00 | 0.00 |
| crmp1=1 AND crmp2=1 AND ybox=1 | 2 / 2 | 1.00 | 1 / 1 | 1.00 | 0.00 |
| crmp1=1 AND crmp2=1 AND ldhb=1 | 2 / 2 | 1.00 | 2 / 2 | 1.00 | 0.00 |

Supplementary Table 3. Abbreviations: ASD, Autism Spectrum Disorders. CRMP1 and CRMP2, collapsin response mediator protein 1 and 2; GDA, guanine deaminase; NSE, neuron specific enolase; LDHA-B, lactate dehydrogenase A and B; STIP1, stress induced phosphoprotein 1 and YBOX, Y-box binding protein 1.

**Supplementary Table 4. All combinations of maternal antibody reactivity to the target autoantigens**

|  | **Training** | | | **Validation** | | **ASD +/ Subgroup** | **Precision** |
| --- | --- | --- | --- | --- | --- | --- | --- |
| ***Single antigen*** | ASD  (n= 206) | TD  (n= 169) | ASD  (n= 244) | | TD (n= 174) |  |  |
| CRMP1 | 8 | 4 | | 3 | 6 | 11/21 | 0.5238 |
| CRMP2 | 1 | 3 | | 2 | 7 | 3/13 | 0.2308 |
| GDA | 15 | 15 | | 9 | 10 | 24/49 | 0.4898 |
| LDHA | 6 | 9 | | 11 | 8 | 17/34 | 0.5000 |
| LDHB | 6 | 5 | | 12 | 6 | 18/29 | 0.6207 |
| NSE | 3 | 6 | | 4 | 1 | 7/14 | 0.5000 |
| STIP1 | 20 | 15 | | 17 | 19 | 37/71 | 0.5211 |
| YBOX | 2 | 3 | | 7 | 4 | 9/16 | 0.5625 |
|  |  |  | |  |  |  |  |
| ***Combination of two antigens*** |  |  | |  |  |  |  |
| **CRMP1 +CRMP2** | **4** | **0** | | **1** | **0** | **5/5** | **1.00** |
| **CRMP1 + GDA** | **6** | **0** | | **3** | **0** | **9/9** | **1.00** |
| **CRMP1 + STIP1** | **4** | **0** | | **0** | **0** | **4/4** | **1.00** |
| **CRMP2 + STIP1** | **1** | **0** | | **1** | **0** | **2/2** | **1.00** |
| **CRMP2 + YBOX** | **1** | **0** | | **0** | **0** | **1/1** | **1.00** |
| **GDA + YBOX** | **1** | **0** | | **1** | **0** | **2/2** | **1.00** |
| **LDHA + YBOX** | **1** | **0** | | **3** | **0** | **4/4** | **1.00** |
| **LDHA + STIP1** | **1** | **0** | | **0** | **0** | **1/1** | **1.00** |
| **LDHB + YBOX** | **1** | **0** | | **0** | **0** | **1/1** | **1.00** |
| **NSE + STIP1** | **3** | **0** | | **2** | **0** | **5/5** | **1.00** |
| **NSE + YBOX** | **0** | **0** | | **1** | **0** | **1/1** | **1.00** |
| STIP1 + YBOX | 2 | 1 | | 3 | 0 | 5/6 | 0.83 |
| GDA + LDHA | 1 | 1 | | 5 | 1 | 6/8 | 0.75 |
| LDHA + LDHB | 3 | 1 | | 4 | 2 | 7/10 | 0.70 |
| CRMP1 + LDHA | 2 | 2 | | 2 | 0 | 4/6 | 0.67 |
| LDHB + STIP1 | 2 | 0 | | 4 | 4 | 6/10 | 0.60 |
| CRMP1 + LDHB | 1 | 1 | | 1 | 1 | 2/4 | 0.50 |
| CRMP2 + LDHA | 1 | 1 | | 1 | 1 | 2/4 | 0.50 |
| LDHA + NSE | 0 | 1 | | 1 | 0 | 1/2 | 0.50 |
| GDA + STIP1 | 3 | 4 | | 2 | 2 | 5/11 | 0.45 |
| GDA + NSE | 3 | 4 | | 2 | 3 | 5/12 | 0.42 |
| LDHA + STIP1 | 2 | 3 | | 1 | 2 | 3/8 | 0.38 |
| GDA + LDHB | 1 | 1 | | 0 | 1 | 1/3 | 0.33 |
| CRMP1 + YBOX | 1 | 1 | | 0 | 2 | 1/4 | 0.25 |
| LDHB + NSE | 1 | 3 | | 0 | 0 | 1/4 | 0.25 |
|  |  |  | |  |  |  |  |
| ***Combination of three antigens*** |  |  | |  |  |  |  |
| **CRMP1 + CRMP2 + LDHB** | **0** | **0** | | **1** | **0** | **1/1** | **1.00** |
| **CRMP1 + CRMP2 + NSE** | **2** | **0** | | **1** | **0** | **3/3** | **1.00** |
| **CRMP1 + CRMP2 + STIP1** | **2** | **0** | | **0** | **0** | **2/2** | **1.00** |
| **CRMP1 + GDA + LDHA** | **2** | **0** | | **0** | **0** | **2/2** | **1.00** |
| **CRMP1 + GDA + LDHB** | **0** | **0** | | **1** | **0** | **1/1** | **1.00** |
| **CRMP1 + GDA + STIP1** | **1** | **0** | | **0** | **0** | **1/1** | **1.00** |
| **CRMP1 + GDA + YBOX** | **1** | **0** | | **0** | **0** | **1/1** | **1.00** |
| **CRMP1 + LDHA + LDHB** | **1** | **0** | | **1** | **0** | **2/2** | **1.00** |
| **CRMP1 + STIP1 + YBOX** | **0** | **0** | | **1** | **0** | **1/1** | **1.00** |
| **CRMP2 + GDA + LDHA** | **1** | **0** | | **0** | **0** | **1/1** | **1.00** |
| **CRMP2 + GDA + NSE** | **1** | **0** | | **0** | **0** | **1/1** | **1.00** |
| **CRMP2 + GDA + STIP1** | **0** | **0** | | **1** | **0** | **1/1** | **1.00** |
| **CRMP2 +LDHA +NSE** | **0** | **0** | | **1** | **0** | **1/1** | **1.00** |
| **GDA + NSE + STIP1** | **1** | **0** | | **0** | **0** | **1/1** | **1.00** |
| **GDA + STIP1 + YBOX** | **1** | **0** | | **0** | **0** | **1/1** | **1.00** |
| **LDHA + STIP1 + YBOX** | **0** | **0** | | **1** | **0** | **1/1** | **1.00** |
| **LDHB + NSE + YBOX** | **1** | **0** | | **1** | **0** | **2/2** | **1.00** |
| **LDHB + STIP1+ YBOX** | **0** | **0** | | **1** | **0** | **1/1** | **1.00** |
| CRMP1 + LDHA + STIP1 | 2 | 1 | | 0 | 0 | 2/3 | 0.67 |
| GDA + LDHA + NSE | 1 | 1 | | 0 | 0 | 1/2 | 0.50 |
| GDA + LDHB + STIP1 | 1 | 0 | | 0 | 1 | 1/2 | 0.50 |
| GDA + LDHA + STIP1 | 1 | 2 | | 1 | 1 | 2/5 | 0.40 |
| CRMP1 + LDHA + NSE | 0 | 1 | | 0 | 1 | 0/2 | 0.00 |
| CRMP1 + LDHB + STIP1 | 0 | 1 | | 0 | 0 | 0/1 | 0.00 |
| GDA + LDHA + LDHB | 0 | 0 | | 0 | 2 | 0/2 | 0.00 |
| LDHA + LDHB + NSE | 0 | 1 | | 0 | 0 | 0/1 | 0.00 |
|  |  |  | |  |  |  |  |
| ***Combination of four antigens*** |  |  | |  |  |  |  |
| **CRMP1+ CRMP2 + GDA + YBOX** | **1** | **0** | | **0** | **0** | **1/1** | **1.00** |
| **CRMP1+ CRMP2 + LDHB + STIP1** | **1** | **0** | | **0** | **0** | **1/1** | **1.00** |
| **CRMP1 + GDA + NSE + STIP1** | **1** | **0** | | **0** | **0** | **1/1** | **1.00** |
| **CRMP1 + LDHB + NSE + STIP1** | **0** | **0** | | **2** | **0** | **2/2** | **1.00** |
| **CRMP2 + NSE + STIP1 + YBOX** | **0** | **0** | | **1** | **0** | **1/1** | **1.00** |
| **GDA + LDHA + NSE + STIP1** | **1** | **0** | | **0** | **0** | **1/1** | **1.00** |
| **LDHA + LDHB + NSE + STIP1** | **0** | **0** | | **1** | **0** | **1/1** | **1.00** |
| CRMP1 + LDHA + LDHB + NSE | 0 | 1 | | 0 | 0 | 0/1 | 0.00 |
|  |  |  | |  |  |  |  |
| ***Combination of five antigens*** |  |  | |  |  |  |  |
| **CRMP1 + GDA + LDHA + LDHB + STIP1** | **1** | **0** | | **0** | **0** | **1/1** | **1.00** |
| **CRMP1 + CRMP2 + LDHA + NSE + STIP1** | **1** | **0** | | **0** | **0** | **1/1** | **1.00** |
| **CRMP1 + CRMP2 + LDHA + LDHB + YBOX** | **0** | **0** | | **1** | **0** | **1/1** | **1.00** |
| **CRMP1 + CRMP2 + GDA + LDHB + STIP1 + YBOX** | **1** | **0** | | **0** | **0** | **1/1** | **1.00** |
| **CRMP1 + GDA + LDHA + LDHB + NSE +STIP1** | **1** | **0** | | **0** | **0** | **1/1** | **1.00** |
|  |  |  | |  |  |  |  |

Supplementary Table 4**.** Abbreviations: ASD, Autism Spectrum Disorders; TD, Typically Developing. CRMP1 and CRMP2, collapsin response mediator protein 1 and 2; GDA, guanine deaminase; NSE, neuron specific enolase; LDHA-B, lactate dehydrogenase A and B; STIP1, stress induced phosphoprotein 1 and YBOX, Y-box binding protein 1.

**Supplementary Table 5. Performance of all the ASD specific patterns found in the training set when tested on the validation set.**

| **Pattern** | **ASD + training** | **ASD + validation** | **Proportion ASD in Validation** | **Proportion of TD in Validation** | **Fisher exact test 2-sided p-value** |
| --- | --- | --- | --- | --- | --- |
| CRMP1 + GDA | 15 | 4 | 1.64% | 0.00% | 0.1443 |
| CRMP1 + CRMP2 | 12 | 4 | 1.64% | 0.00% | 0.1443 |
| STIP1 + NSE | 8 | 6 | 2.46% | 0.00% | 0.0435 |
| STIP1 + CRMP2 | 6 | 3 | 1.23% | 0.00% | 0.2693 |
| YBOX + GDA | 5 | 1 | 0.41% | 0.00% | 1 |
| CRMP2 + NSE | 4 | 3 | 1.23% | 0.00% | 0.2693 |
| CRMP2 + GDA | 4 | 1 | 0.41% | 0.00% | 1 |
| YBOX + CRMP2 | 3 | 2 | 0.82% | 0.00% | 0.5129 |
| STIP1 + CRMP1 + NSE | 3 | 2 | 0.82% | 0.00% | 0.5129 |
| LDHa + STIP1 + NSE | 3 | 1 | 0.41% | 0.00% | 1 |
| LDHb + CRMP1 + GDA | 3 | 1 | 0.41% | 0.00% | 1 |
| CRMP1 + CRMP2 + NSE | 3 | 1 | 0.41% | 0.00% | 1 |
| LDHb + YBOX | 2 | 4 | 1.64% | 0.00% | 0.1443 |
| LDHb + CRMP2 | 2 | 2 | 0.82% | 0.00% | 0.5129 |
| LDHb + CRMP1 + CRMP2 | 2 | 2 | 0.82% | 0.00% | 0.5129 |
| LDHa + LDHb + STIP1 | 2 | 1 | 0.41% | 0.00% | 1 |
| YBOX + CRMP1 + CRMP2 | 2 | 1 | 0.41% | 0.00% | 1 |
| LDHa + YBOX | 1 | 5 | 2.05% | 0.00% | 0.0787 |
| YBOX + NSE | 1 | 3 | 1.23% | 0.00% | 0.2693 |
| LDHb + STIP1 + NSE | 1 | 3 | 1.23% | 0.00% | 0.2693 |
| LDHb + STIP1 + CRMP1 + NSE | 1 | 2 | 0.82% | 0.00% | 0.5129 |
| LDHa + CRMP1 + CRMP2 | 1 | 1 | 0.41% | 0.00% | 1 |
| LDHa + CRMP2 + NSE | 1 | 1 | 0.41% | 0.00% | 1 |
| LDHb + YBOX + STIP1 | 1 | 1 | 0.41% | 0.00% | 1 |
| LDHb + YBOX + CRMP1 | 1 | 1 | 0.41% | 0.00% | 1 |
| LDHb + YBOX + CRMP2 | 1 | 1 | 0.41% | 0.00% | 1 |
| LDHb + YBOX + NSE | 1 | 1 | 0.41% | 0.00% | 1 |
| YBOX + STIP1 + CRMP1 | 1 | 1 | 0.41% | 0.00% | 1 |
| YBOX + STIP1 + CRMP2 | 1 | 1 | 0.41% | 0.00% | 1 |
| STIP1 + CRMP2 + GDA | 1 | 1 | 0.41% | 0.00% | 1 |
| STIP1 + CRMP2 + NSE | 1 | 1 | 0.41% | 0.00% | 1 |
| LDHa + LDHb + STIP1 + NSE | 1 | 1 | 0.41% | 0.00% | 1 |
| LDHb + YBOX + CRMP1 + CRMP2 | 1 | 1 | 0.41% | 0.00% | 1 |
| YBOX + STIP1 | NA | 7 | 2.87% | 0.00% | 0.0448 |
| LDHb + NSE | NA | 4 | 1.64% | 0.00% | 0.1443 |
| STIP1 + CRMP1 | NA | 3 | 1.23% | 0.00% | 0.2693 |
| LDHa + LDHb + CRMP1 | NA | 2 | 0.82% | 0.00% | 0.5129 |
| LDHb + STIP1 + CRMP1 | NA | 2 | 0.82% | 0.00% | 0.5129 |
| LDHb + CRMP1 + NSE | NA | 2 | 0.82% | 0.00% | 0.5129 |
| LDHa + LDHb + YBOX | NA | 1 | 0.41% | 0.00% | 1 |
| LDHa + LDHb + CRMP2 | NA | 1 | 0.41% | 0.00% | 1 |
| LDHa + LDHb + NSE | NA | 1 | 0.41% | 0.00% | 1 |
| LDHa + YBOX + STIP1 | NA | 1 | 0.41% | 0.00% | 1 |
| LDHa + YBOX + CRMP1 | NA | 1 | 0.41% | 0.00% | 1 |
| LDHa + YBOX + CRMP2 | NA | 1 | 0.41% | 0.00% | 1 |
| YBOX + STIP1 + NSE | NA | 1 | 0.41% | 0.00% | 1 |
| YBOX + CRMP2 + NSE | NA | 1 | 0.41% | 0.00% | 1 |
| LDHa + LDHb + YBOX + CRMP1 | NA | 1 | 0.41% | 0.00% | 1 |
| LDHa + LDHb + YBOX + CRMP2 | NA | 1 | 0.41% | 0.00% | 1 |
| LDHa + LDHb + CRMP1 + CRMP2 | NA | 1 | 0.41% | 0.00% | 1 |
| LDHa + YBOX + CRMP1 + CRMP2 | NA | 1 | 0.41% | 0.00% | 1 |
| YBOX + STIP1 + CRMP2 + NSE | NA | 1 | 0.41% | 0.00% | 1 |
| LDHa + LDHb + YBOX + CRMP1 + CRMP2 | NA | 1 | 0.41% | 0.00% | 1 |

Supplementary Table 5. Abbreviations: ASD, Autism Spectrum Disorders; TD, Typically Developing. CRMP1 and CRMP2, collapsin response mediator protein 1 and 2; GDA, guanine deaminase; NSE, neuron specific enolase; LDHA-B, lactate dehydrogenase A and B; STIP1, stress induced phosphoprotein 1 and YBOX, Y-box binding protein 1.

Fisher’s exact (two-sided) was used to evaluate the association of the patterns with ASD diagnosis and p-values > 0.05 were bolded and considered significant.

**Supplementary Table 6. Illustrates autoantibody-antigen reactivity combinations that have over 90% specificity for an ASD diagnosis.**

| ***> 90% ASD SPECIFIC PATTERNS*** | | | | | | |
| --- | --- | --- | --- | --- | --- | --- |
|  | ASD + Training | ASD + Validation | ASD total | ASD % | ASD+/ Subgroup | Precision |
| **STIP1 + YBOX** |  |  |  |  |  |  |
| STIP1 + YBOX | 2 | 3 | 5 | 1.1 | 5/6 | 0.83 |
| CRMP1+ STIP1 + YBOX | 0 | 1 | 1 | 0.2 | 1/1 | 1.00 |
| GDA + STIP1 + YBOX | 1 | 0 | 1 | 0.2 | 1/1 | 1.00 |
| LDHA + STIP1 + YBOX | 0 | 1 | 1 | 0.2 | 1/1 | 1.00 |
| LDHB + STIP1+ YBOX | 0 | 1 | 1 | 0.2 | 1/1 | 1.00 |
| CRMP2 + NSE + STIP1 + YBOX | 0 | 1 | 1 | 0.2 | 1/1 | 1.00 |
| CRMP1 + CRMP2 + GDA + LDHB + STIP1 + YBOX | 1 | 0 | 1 | 0.2 | 1/1 | 1.00 |
| **Total** | **4** | **7** | **11** | **2.4** | **11/12** | **0.92** |
|  |  |  |  |  |  |  |
| **CRMP1 + STIP1** |  |  |  |  |  |  |
| CRMP1 + STIP1 | 4 | 0 | 4 | 0.9 | 4/4 | 1.00 |
| CRMP1 + LDHA + STIP1 | 2 | 0 | 2 | 0.4 | 2/3 | 0.67 |
| CRMP1 + CRMP2 + STIP1 | 2 | 0 | 2 | 0.4 | 2/2 | 1.00 |
| CRMP1 + LDHB + STIP1 | 0 | 0 | 0 | 0.0 | 0/1 | 0.00 |
| CRMP1 + GDA + STIP1 | 1 | 0 | 1 | 0.2 | 1/1 | 1.00 |
| CRMP1+ STIP1 + YBOX | 0 | 1 | 1 | 0.2 | 1/1 | 1.00 |
| CRMP1 + GDA + NSE + STIP1 | 1 | 0 | 1 | 0.2 | 1/1 | 1.00 |
| CRMP1 + LDHB + NSE + STIP1 | 0 | 2 | 2 | 0.4 | 2/2 | 1.00 |
| CRMP1+ CRMP2 + LDHB + STIP1 | 1 | 0 | 1 | 0.2 | 1/1 | 1.00 |
| CRMP1 + GDA + LDHA + LDHB + STIP1 | 1 | 0 | 1 | 0.2 | 1/1 | 1.00 |
| CRMP1 + CRMP2 + LDHA + NSE + STIP1 | 1 | 0 | 1 | 0.2 | 1/1 | 1.00 |
| CRMP1 + GDA + LDHA + LDHB + NSE +STIP1 | 1 | 0 | 1 | 0.2 | 1/1 | 1.00 |
| CRMP1 + CRMP2 + GDA + LDHB + STIP1 + YBOX | 1 | 0 | 1 | 0.2 | 1/1 | 1.00 |
| **Total** | **15** | **3** | **18** | **4.0** | **18/20** | **0.9** |
|  |  |  |  |  |  |  |

Supplementary Table 6. Summary of 90% specific ASD patterns. Abbreviations: ASD, Autism Spectrum Disorders; TD, Typically Developing. CRMP1 and CRMP2, collapsin response mediator proteins 1 and 2; GDA, guanine deaminase; NSE, neuron specific enolase; LDHA-B, lactate dehydrogenase A and B; STIP1, stress induced phosphoprotein 1 and YBOX, Y-box binding protein 1.
